# The role of smartphones in adolescent-parent discrepancy in reporting adolescents’ internalizing problems

**DOI:** 10.1017/S0954579425100618

**Published:** 2025-09-08

**Authors:** Cory Carvalho, Kalsea Koss, Niyantri Ravindran

**Affiliations:** Human Development and Family Science, University of Georgia, Athens, USA

**Keywords:** Adolescence, family technology, internalizing problems, parent-child informant discrepancy, parent-child relationships, smartphones

## Abstract

The current study examined how early smartphone ownership impacts parent-child informant discrepancy of youth internalizing problems during the transition to adolescence. We used four waves of longitudinal data (Years 1–4) from the Adolescent Brain Cognitive Development (ABCD; Baseline *N* = 11,878; White = 52.0%, Hispanic = 20.3%, Black = 15.0%, Asian = 2.1%, Other = 10.5%; Female = 47.8%). Across the full sample, significant parent-child informant discrepancy, such that parents underestimated child reports, appeared at Year 2 (*M*_*age*_ = 12.0) and increased across the remainder of the study (*b* = −0.21, *SE* = .042, *p* < .001, 95%CI [−.29, −.23]). Further, multi-group models indicated that significant parent-child informant discrepancy emerged in the years following initial smartphone acquisition, whereas youth who remained non smartphone owners did not demonstrate such a pattern. Moreover, this discrepancy grew with additional years of smartphone ownership. This study contributes to the ongoing discourse on adolescent smartphone use and mental health by documenting a novel, longitudinally observed risk to timely parental detection of mental health problems by early smartphone ownership.

## Introduction

The release of the iPhone in 2007 marked a turning point in social interactions. Since then, adolescent smartphone ownership has steadily increased, with 43% of 8 – 12 year olds and 88% of 13 – 18 year olds owning smartphones by 2021 (Rideout & Robb, 2020). This rapid adoption has prompted research examining its impact on the parent-child relationship (Hawi & Samaha, 2017). Smartphone use can reduce face-to-face communication, fragment attention, and isolate one from their immediate physical and social environment (Dwyer et al., 2018). Empirical evidence suggests that smartphones can degrade communication quality and compromise functioning in close relationships (McDaniel, 2014; Sbarra et al., 2019). This impact on parent-child relationships may lead to fewer conversations about youths’ mental health, potentially impacting parent and youth reporting agreement of youth internalizing problems (Kim et al., 2016; Robinson et al., 2019; Yang et al., 2021). Notably, early adolescence marks not only when smartphone ownership begins but also when internalizing problems, such as anxiety and depression, become more prevalent (Thapar et al., 2012). Moreover, meta-analyses indicate that parent-adolescent informant discrepancy is more pronounced for internalizing problems than other types psychopathology (De Los Reyes et al., 2015). Parents tend to underestimate youth-reported internalizing symptoms, as they are less observable and often rely on parental solicitation or youth disclosure for detection (Kapetanovic et al., 2020; Makol & Polo, 2018). This raises the question: Does owning a smartphone in adolescence increase the likelihood of parent-child discrepancy in reporting internalizing problems? This is a critical concern because parents’ awareness of their children’s mental health is essential for timely intervention. Moreover, parent-child informant discrepancy of psychopathology is a known developmental risk factor, predisposing youth to impaired social functioning and mental health problems (Castagna et al., 2021; Fabris et al., 2020; Goolsby et al., 2018; Koca & Saatçı, 2022). Therefore, the present study aimed to examine the role of adolescent smartphone ownership in parent-child reporting discrepancy of youth internalizing problems.

### Smartphone use in adolescence

Youth typically become smartphone owners between the age of 8 (31%) and 14 (72%) according to Rideout et al. (2022). Smartphones present adolescents with a unique developmental challenge. Neurobiologically, they experience significant changes that lead to increased novelty-seeking and risk-taking (Steinberg, 2007), more autonomy from parents (Spear & Kulbok, 2004), and greater reliance on peers for social support (Farley & Kim-Spoon, 2014). At the same time, they experience trailing development in prefrontal brain regions that support executive control and emotion regulation (Romer et al., 2017). Concurrently, various addiction (Poudel & Gautam, 2017) and mental health problems (Thapar et al., 2012) tend to first emerge in adolescence as youth renegotiate their place among their peers and the broader social milieu. Amidst this sociobiological backdrop, smartphones present endless opportunities for social and entertainment stimulation. Further increasing their appeal, tech platforms present content through smartphone apps that are algorithmically designed to maximize the capture of users’ attention. Consequently, these smartphone qualities can negatively impact close relationships (Sbarra et al., 2019), potentially interfering with parental detection of fluctuations in their youths’ mental health status.

### Parent-child discrepancy in reporting adolescent psychosocial health problems

Using multiple informant methods to measure youth mental health is recognized as a strength in research and clinical practices (De Los Reyes et al., 2015). This is because parents and children sometimes report different yet valid information based on their unique perspectives (Karver, 2006). However, empirical studies suggest the concordance between parent’s and child’s reports are typically low to moderate in adolescence across a range of psychosocial outcomes (Rescorla, 2016), particularly for internalizing problems (De Los Reyes et al., 2015). Low rates of agreement partly reflect excessive reporting discrepancy and may indicate parents’ lack of awareness of their children’s mental health status (Aebi et al., 2017; Lagattuta et al., 2012). Moreover, parents’ sensitivity to their children’s changing mental health is important for making remedial adjustments within the family and seeking early intervention (Goolsby et al., 2018) and greater informant discrepancy has been identified as a risk factor for youth well-being (Castagna et al., 2021). However, longitudinal studies assessing trajectories of informant discrepancy in reporting internalizing problems across early adolescence are scarce.

Various contributing factors to higher parent-child reporting discrepancy have been identified (Kolko & Kazdin, 1993; Treutler & Epkins, 2003), including relationship quality (Kim et al., 2016; Robinson et al., 2019; Yang et al., 2021). For example, poorer parental engagement (Van Roy et al., 2010), less communication (Barker et al., 2007; Van Roy et al., 2010), weaker maternal bonding (Chen et al., 2017), and lower parental monitoring (Laird & LaFleur, 2016) have been associated with stronger disagreement. Further, qualitative studies suggest that discrepancy can emerge when parents are unaware of adolescents’ symptoms or misread their behavior (Bidaut-Russell et al., 1995).

### Smartphones and parent-child discrepancy

Smartphones appeal to parents and youth partly because they allow dyads the ability to communicate despite changes in proximity (Richter et al., 2022). This aspect of smartphone ownership offers clear benefits as adolescents enjoy increasing autonomy and time separated from their parents (Zimmer-Gembeck & Collins, 2006). With smartphones, parents can more easily monitor youths’ whereabouts (Weisskirch, 2009) and youth can conveniently convey information to their parents about changes in plans or safety concerns (Warren & Aloia, 2018).

Smartphones also have the potential to negatively impact the quality of interactions in close relationships (Sbarra et al., 2019), including in the parent-child dyad (Stockdale et al., 2018). As adolescents increasingly spend time with smartphones, face-to-face interactions can degrade in frequency and quality within the home (McDaniel, 2014) and feelings of being distant increase between parents and youth (Lanette, 2018). In fact, the mere presence of a smartphone has been shown to decrease feelings of connectedness between individuals in proximity (Misra et al., 2016; Przybylski & Weinstein, 2013). Moreover, Davis et al. (2019) found evidence supporting the theory that excessive adolescent smartphone use contributes to parent-teen disconnectedness.

Given the impact of smartphones on close relationships, youths’ access to smartphones may contribute to informant discrepancy in reporting internalizing problems between parents and adolescents. Smartphones have been shown to possess addictive qualities that capture youths’ attention (Yildirim et al., 2024), potentially reducing the frequency and quality of parent-child interactions. Reduced engagement provides fewer opportunities for parental solicitation and observation of affective cues. Moreover, even during interactions, smartphone use may fragment attention and diminish interaction quality (Lanette, 2018), further limiting parents’ ability to perceive internalizing cues. Consequently, adolescents’ preoccupation with smartphones may hinder parents’ awareness of their children’s internalizing problems.

Smartphone use may also decrease adolescents’ disclosure of internalizing problems to their parents. Social media, the most prominent smartphone activity, accounts for more than twice the daily usage time compared to other activities (Alexander et al., 2024). By expanding the peer social environment beyond schools, extracurricular activities, and social outings to virtually all locations, including the home, social media blurs the boundaries between peer interactions and family time. Previously, adolescents could separate socializing with peers from debriefing with parents at home. Now, they carry their entire social network in their pockets (Kushlev et al., 2019). This constant access enables adolescents to share emotional burdens with peers at any time of day. Although peer support has its benefits, unrestricted access to peer networks may reduce adolescents’ reliance on parental support, leaving parents less informed about their child’s internalizing problems and the contextual factors influencing it.

Youth are acquiring smartphones at increasingly younger ages, and early smartphone ownership has been linked to a range of maladaptive developmental outcomes (Sapien Labs, 2023). Given evidence that smartphones can disrupt close relationships (Lanette, 2018; Sbarra et al., 2019), early smartphone ownership may contribute to parent-child informant discrepancy regarding youth internalizing problems. Specifically, earlier smartphone adoption could influence parent-child relational dynamics during a period of heightened psychological vulnerability due to normative developmental changes. Therefore, it is essential to investigate whether early smartphone ownership is associated with parent-child discrepancy in reporting youth internalizing problems across early adolescence.

### The current study

Trajectories of parent-child discrepancy in reporting of youth internalizing problems are understudied in early adolescent youth. Moreover, smartphones can diminish the frequency and quality of interactions in close relationships (Sbarra et al., 2019), potentially limiting opportunities for child disclosure and parental solicitation of internalizing concerns. This could increase parent-child reporting discrepancy of youth internalizing problems. However, the role of early smartphone ownership in parent-child informant discrepancy has not yet been examined. To address this gap, we used a multi-level framework to analyze smartphone ownership age and parent- and child-reported internalizing problems using data from the ABCD Study (Y1: *M*_*age*_ = 10.9, Y2: *M*_*age*_ = 10.9, Y3: *M*_*age*_ = 12.9 Y4: *M*_*age*_ = 14.1). This approach is recommended for modeling longitudinal informant discrepancy as an outcome (De Los Reyes et al., 2016) as it addresses the limitations of difference scores analyses (see Edwards, 1994; Laird & LaFleur, 2016 for review). First, we examined average changes in discrepancy over time to show the general trajectory of parent-adolescent informant discrepancy across early adolescence in the whole sample. We hypothesized that informant discrepancy would increase across early adolescence. Then, we tested whether overall parent-child reporting discrepancy of youth internalizing problems (i.e., averaged across all time points) varies by age of smartphone ownership across early adolescence after controlling for parental education, youth biological sex, stage of pubertal development, and overall screen time. We hypothesized that discrepancy, such that parents underestimate youths’ internalizing problems, would be higher with earlier smartphones ownership. Lastly, we examined changes in informant discrepancy over time based on the age of onset of youth smartphone ownership. We hypothesized there would be a significant moderating effect of time on informant discrepancy among youth who own smartphones. Further, we hypothesized that the informant effects would be more pronounced in each group for the measurement occasions after youth receive a smartphone. If supported, this would suggest that smartphone ownership contributes to increasing parent-child reporting discrepancy across early adolescence implying smartphone impacts on parent-child relationship dynamics.

## Method

We used data from the Adolescent Brain Cognitive Development (ABCD) study to address study aims. The ABCD study recruited 11,876 adolescents aged 9 – 10 at baseline and their primary caregivers beginning in 2015 with follow-up data collection planned every 6 months for a total of 10 years. Overall, data collected consists of neuroimaging, cognitive-tasks, and survey data. Data for the current study was from the 5.1 release which includes approximately half of the data from the most recent wave (i.e., Year 4 in the current study) according to the ABCD Study’s planned missingness data release design.

### Participants

For the current study, survey data from complete parent-adolescent dyads were used from the baseline (Y0: *N* = 11,876; *M*_*age*_ = 9.9), one-year follow-up (Y1: *N* = 11,219; *M*_*age*_ = 10.9), two-year follow-up (Y2: *N* = 10,972; *M*_*age*_ = 12.0), three-year follow-up (Y3: *N* = 10,335; *M*_*age*_ = 12.9), and four-year follow-up (Y4: *N* = 4,754; *M*_*age*_ = 14.1). The sample was designed to be a representative sample of US adolescents (White = 52.0%, Hispanic = 20.3%, Black = 15.0%, Asian = 2.1%, Other = 10.5%; Female = 47.8%). Parent surveys were completed mostly by biological mothers (85.3%; 10.0% biological fathers; 2.3% adoptive parents; 1.0% custodial parent; 1.4% other) and participants were instructed to include the same parent at each measurement occasion.

### Measures

#### Age of smartphone ownership

Parents were asked several questions regarding their child’s smartphone ownership. First, they were asked whether or not their child owns a smartphone. If parents responded “No” to this item at Y4, then youth were assigned a value of “5” representing no smartphone ownership. In addition, parents were asked annually at what age did their children first own a cell phone. If answers changed throughout the study, then the earlier response was used. Then, smartphone ownership data was recoded according to the following categories based on youths age at each measurement occasion: 8 & under, 9 & 10, 11 & 12, 13 & 14, and No Phone.

#### Child internalizing problems (parent- and youth-reported)

Internalizing problems were reported by parents using the 112-item Child Behavior Checklist (CBCL; Achenbach & Edelbrock, 1991) and by youth using the 18-item Brief Problem Monitor (BPM; Achenbach et al., 2011), which is a companion to the CBCL. The BPM was first administered at the one-year follow-up wave. For each scale, reporters responded to symptoms of internalizing problems according to a 3-item Likert format (0 = Not true, 1 = Somewhat/ Sometimes true, 2 = Very true). To model discrepancies, internalizing problems for the current study are limited to the six items that are present in both the BPM and the CBCL. These shared items are characterized as anxious/depressed (3 items) or withdrawn/depressed (3 items) aspects of internalizing problems (see Supplemental Table 1 for specific items) according the scale’s syndrome scoring protocol (Achenbach et al., 2011). Internal reliabilities for these six items were acceptable at each timepoint and higher for parents (α_1_ = .756; α_2_ = .762; α_3_ = .776; α_4_ = .783) than youth (α_1_ = .619; α_2_ = .615; α_3_ = .619; α_4_ = .616). Internalizing problems distributions were right skewed consistent with prevalence rates for internalizing problems during adolescence; however, observed data span the full range of possible scores (Min = 0, Max = 12) represented at each timepoint for each reporter (see Supplemental Figure 1 for distributions).

#### Covariates

Parental education and youth biological sex were included as between-level control variables. Parental education level was operationalized as: (1) Did not graduate high school, (2) High school diploma or GED, (3) Some college/Associate’s degree; (4) Bachelor’s degree; and (5) Graduate degree. Further, the effects of pubertal development and overall screentime were modeled at the within-level as time-varying covariates as well as at the between-level by regressing internalizing problems on the respective person-level means. Pubertal development was reported by parents at each time point using the Pubertal Development Scale (Cheng et al., 2021; Petersen et al., 1988). Parents responded to five items regarding various physical changes related to puberty (e.g., growth spurts and body hair) on a 4-point Likert scale from “has not begun” (1) to “seems complete” (4). Two of the five items were specific to boys or girls, respectively. An average value for each timepoint was used in analyses. Youth reported their screentime via four items from the Screen Time Questionnaire (Bagot et al., 2022) targeting their total weekday and weekend hours and minutes screen usage for any non-educational purpose. A weighted mean (i.e., 2/7 weekend + 5/7 weekday) was calculated to represent their average total screentime. Values were winsorized to ± 3SD from the mean. Youth-reported screentime has been demonstrated to be more reliable than parents in an ABCD Study subsample (Wade et al., 2021) when compared against objective passive sensing measures (*N* = 67, Youth *r* = .49, *p* < .001, Parent *r* = .10, *p* = .43).

### Analytic plan

All study analyses were conducted using Mplus version 8.3. Attrition remained modest for longitudinal variables through Y3 (∼15% drop from baseline for parent- and youth-reported internalizing problems and 17.9% for smartphone ownership age). Analyses of missing data patterns revealed that boys (*F* = 10.4, *p* = .001) and youth from parents with lower education (*F* = 154.2, *p* < .001) were more likely to drop out after Y1. These variables were included as covariates and thus missing data were treated as Missing at Random (MAR). Additionally, consistent with ABCD’s data release strategy, roughly half the sample had Y4 data, and all available data were included in analyses with the unreleased data estimated and assumed MAR as this release strategy is consistent with planned missingness designs. Thus, missing data were handled using full information maximum likelihood with robust standard errors (MLR) estimation because it produces unbiased parameter estimates with non-normal data (Yuan & Bentler, 2000). First, means and correlations among study variables were examined to assess overall sample characteristics and bivariate associations at each time point.

Second, we tested the overall and change in informant discrepancy for the whole sample.

Empirical studies often use *X* – *Y* difference scores (e.g., simple, absolute, standardized) to evaluate reporting discrepancies. However, critiques have identified limitations that undermine the validity of this approach (Edwards, 1994; Laird & De Los Reyes, 2013). For example, if reporters demonstrate unequal variance across the sample (e.g., parents vs. youth), then the results are driven primarily by the reporter with more variability (Edwards, 1994). Second, difference scores often artificially reduce variance compared to their components which can reduce statistical power. Third, because they are a composite of two values they inherit the accumulation of measurement error from both values, decreasing reliability (Edwards, 1994). Fourth, difference scores introduce ambiguity when interpreting effects because the same difference score can result from various combinations of *X* and *Y* (Laird & De Los Reyes, 2013). As an alternative, polynomial regression (i.e., for cross-sectional models) and multi-level modeling (i.e., for longitudinal models) have been shown to be superior (De Los Reyes et al., 2016) and were used in the current study as described in the following paragraphs.

Thus, we conducted a series of longitudinal models to examine whether significant parent-child informant discrepancy in reporting youths’ internalizing problems was present in the sample and whether this discrepancy changed across time. We used multi-level modeling to distinguish between-dyad variance from within-dyad variance across time as is recommended by De Los Reyes et al. (2016). Internalizing problems were modeled as the outcome variable with informant (i.e., child = 0 and parent = 1), Time, and the Time × Informant product modeled as predictors. In building the model, we first determined in the full sample whether fixed or random effects for Time and Informant provided the better fit using the Satorra-Bentler scaled chi-square difference test (Satorra & Bentler, 2001). This test is used in place of the standard likelihood ratio test when MLR estimation is used due to the presence of non-normality. Then, in the first model, we tested the within-level direct effects of Time and Informant on internalizing problems. Pubertal development and overall screen time at each wave were included at the within-person level as a time-varying fixed effects while youth sex and parental education were included as time-invariant covariates at the between-level. Then, we added the Time × Informant product term at the within-level to test whether informant discrepancy changed across time. In this final model, *β*_0i_ represents the mean of child’s internalizing problems when time is zero (e.g., the one-year follow-up) and the informant is the child, *β*_1i_ represents the effect of Time for individual *i* at time *j, β*_2i_ represents the effect of informant *k* on individual *i* (i.e., parent-child informant discrepancy), and *β*_3i_ represents the moderating effect of time *i* on the effect of informant *k* on internalizing problems. In this model, observations are nested within individuals where time *j* and informant *k* are level 1 predictors.

Level 1:

Internalizing Problems_ijk_ = *β*_0i_ + *β*_1i_(Time_ij_) + *β*_2i_(Informant_k_) + *β*_3i_(Time_ij_ × Informant_k_) + ε_ijk_

Level 2:

*β*_0i_ = γ_0_ + u_0i_

*β*_1i_ = γ_1_ + u_1i_

*β*_2i_ = γ_2_ + u_2i_

*β*_3i_ = γ_3_ + u_3i_

Next, we conducted the above outlined procedure in multi-group models based on age of smartphone ownership to determine whether the informant effects varied across these smartphone age-based groups. We then probed the interactions to further examine how informant discrepancy changed across time for each group. Propensity weights provided by the ABCD Study were also accounted for in all models to calibrate distributions to nationally representative controls based on the American Community survey to increase the generalizability of findings (see Heeringa & Berglund, 2020; Saragosa-Harris et al., 2022 for more information).

## Results

### Descriptive statistics

Means, standard deviations, and correlations among study variables for the whole sample are displayed in Table 1. Parental education was positively correlated with age of cellphone ownership (*r* = .23, *p* < .001), indicating that youth with more educated parents tended to acquire smartphones at older ages. Girls generally obtained smartphones earlier than boys (*r* = .07, *p* < .001). Internalizing problems increased across the four measurement occasions, with youth consistently reporting higher levels than their parents at each time point.

**Table 1.** Means, standard deviations, and correlations of study variables

Variable	1	2	3	4	5	6	7	8	9	10	11
1. Internal (Y) 1	–										
2. Internal (Y) 2	.51***	–									
3. Internal (Y) 3	.40***	.58***	–								
4. Internal (Y) 4	.35***	.44***	.58***	–							
5. Internal (P) 1	.25***	.23***	.20***	.18***	–						
6. Internal (P) 2	.22***	.30***	.26***	.24***	.65***	–					
7. Internal (P) 3	.21***	.30***	.34***	.27***	.59***	.67***	–				
8. Internal (P) 4	.23***	.28***	.31***	.38***	.54***	.61***	.67***	–			
9. Youth sex	−.03*	−.15***	−.23***	−.29***	.00	−.04**	−.07***	−.14***	–		
10. Education (P)	−.09***	−.06***	−.03*	−.01	.06***	.08***	.09***	.08***	.01	–	
11. Cellphone age	−.04**	−.04**	−.07***	−.06***	.03**	.04**	.04**	.03	.07***	.23***	–
*M*	1.74	1.81	2.02	2.27	1.49	1.43	1.46	1.48	.52	3.74	2.72
*SD*	2.11	2.23	2.38	2.56	1.88	1.88	1.93	1.96	.50	1.17	0.85

*Note. Y* = Youth-report; *P* = Parent-report; Internal = Internalizing problems. ***p < .001, **p < .01, *p < .05.

Table 2 shows the size, proportional demographics, and mean internalizing problems for each cellphone age group. Most youth were in the 9&10 (*N* = 2,641) and 11&12 (*N* = 5,037) groups while less membership was found in the 8 & under (*N* = 677), 13&14 (*N* = 744), and no phone (*N* = 376) groups. There was similar representation between boys and girls in the early smartphone ownership groups (8 & under: 53% girls, χ^2^ = 2.77, df = 1, *p* = .096; 9&10 : 51% girls, χ^2^ = 2.47, df = 1, *p* = .116) but more boys in the remaining groups (11&12: 48% girls, χ^2^ = 7.55, df = 1, *p* = .006; 13&14: 39% girls, χ^2^ = 31.15, df = 1, *p* < .001; No Phone: 37% girls, χ^2^ = 23.50, df = 1, *p* > .001). Youth with parents who had higher education tended to be proportionally larger in groups in which youth began smartphone ownership later or did not yet own a smartphone. ANOVA tests revealed significant differences across groups on all variables in Table 2 (Parent education: *F* = 146.40, df = 4, *p* < .001; Youth-reported internalizing problems: Y2 *F*_*min*_ = 26.08, *p* < .001, Y4 *F*_*max*_ = 65.43, df = 4, *p* < .001; Parent-reported internalizing problems: Y1 *F*_*min*_ = 14.84, *p* = .002, Y2 *F*_*max*_ = 20.13, df = 4, *p* < .001), except for parent-reported internalizing problems at Y4 (*F* = 8.69, df = 4, *p* = .060).

**Table 2.** Group means for internalizing problems across time and demographic covariates

	8 & under	9 − 10	11 − 12	13 − 14	No phone
*N*	677	2641	5037	744	376
	***M*(*SD*)**	***M*(*SD*)**	***M*(*SD*)**	***M*(*SD*)**	***M*(*SD*)**
Sex (0 = F, 1 = M)	.47(.50)	.49(.50)	.52(.50)	.61(.49)	.63(.49)
Parent education	3.06(1.10)	3.51(1.15)	3.89(1.13)	4.10(0.99)	4.19(1.01)
	***M*(*SD*)**	***M*(*SD*)**	***M*(*SD*)**	***M*(*SD*)**	***M*(*SD*)**
Youth Y1 (10 − 11)	2.03(2.23)	1.82(2.11)	1.71(2.11)	1.46(1.91)	1.85(2.23)
Youth Y2 (11 − 12)	2.05(2.43)	1.95(2.26)	1.79(2.21)	1.62(2.15)	1.80(2.31)
Youth Y3 (12 − 13)	2.36(2.56)	2.16(2.48)	2.07(2.40)	1.75(2.14)	1.50(1.96)
Youth Y4 (13 − 14)	2.56(2.72)	2.36(2.67)	2.31(2.57)	2.04(2.40)	1.82(2.17)
	***M*(*SD*)**	***M*(*SD*)**	***M*(*SD*)**	***M*(*SD*)**	***M*(*SD*)**
Parent Y1 (10 − 11)	1.49(2.00)	1.39(1.82)	1.52(1.87)	1.50(1.84)	1.75(2.00)
Parent Y2 (11 − 12)	1.30(1.94)	1.35(1.79)	1.45(1.88)	1.44(1.89)	1.80(2.21)
Parent Y3 (12 − 13)	1.34(1.99)	1.36(1.86)	1.52(1.99)	1.44(1.77)	1.72(2.14)
Parent Y4 (13 − 14)	1.31(1.81)	1.39(1.89)	1.54(2.01)	1.40(1.80)	1.60(2.21)

*Note. F* = female; *M* = male.

For internalizing problems, youth reported higher levels of internalizing problems than parents at all time points, except for the No Phone group. Also, in each of these groups, youth reported higher levels of internalizing problems at each consecutive year. In contrast for youth who did not own a smartphone, youth reported higher levels of internalizing problems at Y1 and Y4, parents and youth reported the same levels at Y2, and parents reported higher levels at Y3. Notably, after Y1, although youth reported higher mean levels of internalizing problems with earlier smartphone ownership, at the same time their parents generally reported lower mean levels, respectively.

**Figure 1.**
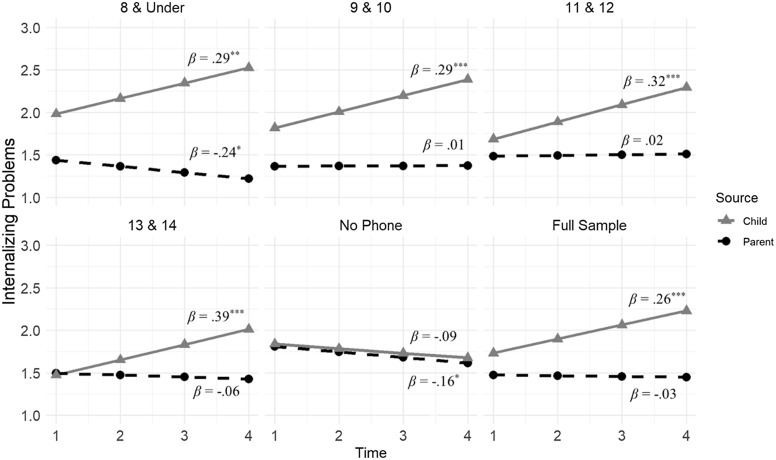
Multi-group latent growth curve analysis of parent- and child-reported youth internalizing problems by age of cell phone ownership. *Note.* ***p < .001, **p < .01, *p < .05.

A post-hoc multi-group latent growth curve analysis (see Figure 1) revealed that youth-reported internalizing problems showed significant positive slopes over time in all groups with the exception of the No Phone group (8 & under: *b* = .39, *β* = .29, *SE* = .12, *p* = .001; 9&10: *b* = .19, *β* = .29, *SE* = .02, *p* < .001; 11&12: *b* = .20, *β* = .32, *SE* = .02, *p* < .001; 13&14: *b* = .21, *β* = .39, *SE* = .06, *p* < .001; No Phone: *b* = −.05, *β* = −.09, *SE* = .04, *p* = .216). However, parent-reported internalizing problems showed significant negative slopes in the 8 & under (*b* = −.07, *β* = −.24, *SE* = .03, *p* = .013) and No Phone (*b* = −.07, *β* = −.19, *SE* = .03, *p* = .044) groups, yet non-significant growth in the remaining groups (9&10: *b* = .00, *β* = .01, *SE* = .01, *p* = .858; 11&12: *b* = .01, *β* = .02, *SE* = .02, *p* = .439; 13&14: *b* = −.02, *β* = −.06, *SE* = .02, *p* = .308). Further, within-time correlations between parent and child reports of internalizing problems increased across the three years in the full sample (Y1: *r* = .25, *p* < .001; Y2: *r* = .30, *p* < .001; Y3: *r* = .34, *p* < .001; Y4: *r* = .38, *p* < .001).

### Multi-level parent-child reporting discrepancy of internalizing problems models

#### Full sample random and fixed effects comparison

**Figure 2.**
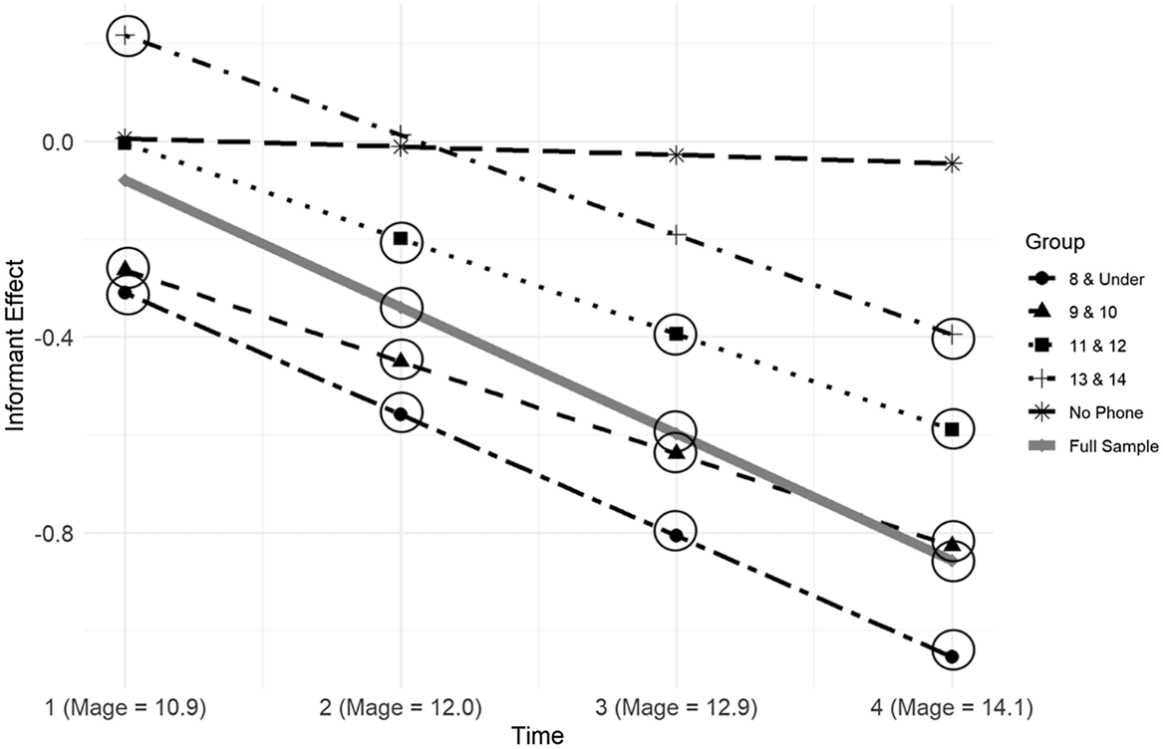
Predicted values of informant effect by age of smartphone ownership group. *Note*. A negative informant effect indicates parent-report underestimated youth-report. Significant informant effects are indicated with circles (*p* < .05).

The Satorra-Bentler scaled chi-square difference test was conducted to compare the fixed effects and random effects models of the full sample. Test results showed that modeling random effects significantly improved the model over fixed effects (χ_SB_^2^(2) = 8753.51, *p* < .001). After controlling for parent education, youth sex, and within-level pubertal development, there were significant effects of time (*b* = 0.04, *SE* = 0.01, *p* < .001) and informant (Youth = 0, Parent = 1; *b* = −0.47, *SE* = .02, *p* < .001) on internalizing problems across the full sample. This suggests that overall (i.e., parent- and self-reports collectively) internalizing problems increased across the four-wave period and that youth reported higher levels on average. Further, there was a significant negative effect of the product between time and informant on internalizing problems (*b* = −0.21, *SE* = .042, *p* < .001, 95%CI [−.29, −.23]), suggesting informant discrepancy increased with youth age across the whole sample. Probing the interaction effect (see Figure 2) revealed a significant negative informant effect (Youth = 0, Parent = 1) at baseline (Y1: *M*_*age*_ = 10.9, *b* = −.08, *SE* = .03, *p* = .009) that increased across all subsequent timepoints (Y2: *M*_*age*_ = 10.9, *b* = −.34, *SE* = .03, *p* < .001; Y3: *M*_*age*_ = 12.9, *b* = −.60, *SE* = .04, *p* < .001; Y4: *M*_*age*_ = 14.1, *b* = −.86, *SE* = .06, *p* < .001).

#### Multi-group direct effects of time and informant on youth internalizing problems

Parameter estimates for the direct effects of time and informant on youth internalizing problems are presented in Table 3. For within-level covariate effects, total screen time was related to internalizing problems in the 8 & under, 9&10, and 11&12 groups, but not in the 13&14 and No Phone groups. Moreover, pubertal development was related to internalizing problems in only the 9&10 group. For between-level covariate effects, girls reported significantly more overall internalizing problems in all groups, except for the No Phone group. Moreover, higher parent education was related to more overall internalizing problems in the 9&10 group and 11&12 groups, but not in the 8 & under, 13&14, and No Phone groups. Further, mean person-level total screen time was related to more internalizing problems in all groups, except for 8&under.

**Table 3.** Parameter estimates for multi-level models examining the impact of time, informant, and time × informant on internalizing problems based on age of smartphone ownership

	8 & under			9 & 10			11 & 12			13 & 14			No Phone		
**Level 1**	***Est***	***SE***	***p***	***Est***	***SE***	***p***	***Est***	***SE***	***p***	***Est***	***SE***	***p***	***Est***	***SE***	***p***
**Covariates**															
Puberty → IP	.08	.06	.179	.09	.03	< .001	.02	.02	.249	.08	.04	.073	.06	.07	.401
Screen time → IP	.05	.01	< .001	.02	.01	< .001	.03	.01	< .001	.02	.01	.051	.03	.02	.064
**Random effects**															
β_1_ Time → IP	.03	.03	.426	.06	.02	.002	.09	.01	< .001	.03	.03	.276	.06	.07	.401
β_2_ Informant → IP	−.85	.08	< .001	−.68	.04	< .001	−.44	.03	< .001	−.27	.06	< .001	.03	.02	.064
β_3_ Time × Inf. → IP	−.25	.05	< .001	−.19	.02	< .001	−.20	.02	< .001	−.20	.04	< .001	−.02	.05	.747
**Level 2**	***Est***	***SE***	***p***	***Est***	***SE***	***p***	***Est***	***SE***	***p***	***Est***	***SE***	***p***	***Est***	***SE***	***p***
**Covariates**															
Youth sex → IP_mean_	−.41	.15	.005	−.29	.07	< .001	−.32	.05	< .001	−.36	.13	.007	−.23	.22	.298
Pedu → IP_mean_	.08	.05	.160	.11	.02	< .001	.09	.02	< .001	.08	.05	.112	.03	.07	.693
PD_mean_ → IP_mean_	.02	.10	.868	.03	.04	.424	.10	.03	.002	.14	.09	.111	.04	.13	.780
ST_mean_ → IP_mean_	.02	.02	.294	.06	.01	< .001	.12	.01	< .001	.13	.03	< .001	.09	.04	.037

*Note.* IP = Internalizing problems, Pedu = Parent’s education, PD = Pubertal development, ST = Screen time.

Direct effects of time (i.e., wave of data collection) and informant (i.e., youth = 0 or parent = 1) are shown in Table 3 prior to adding the interaction (i.e., time × informant) to the model. Time was positively related to internalizing problems for all current cellphone ownership groups, though only significantly for 9&10 and 11&12 (8 & under: *b* = .03, *SE* = .03 *p* = .426; 9 – 10: *b* = .06, *SE* = .02, *p* = .002; 11 – 12: *b* = .09, *SE* = .01, *p* < .001, 13 – 14: *b* = .03, *SE* = .03, *p* = .276). This suggests that the combined average parent- and youth-reported internalizing problems increased across the 3-year period for all groups in which youth owned a smartphone. In contrast, a negative, non-significant association between time and internalizing problems was found in the No Phone group (*b* = −.09, *SE* = .05, *p* = .068).

For overall informant discrepancy, a pattern emerged in which the negative effect of informant (Youth = 0, Parent = 1) on average internalizing problems across all timepoints was increasingly more pronounced the earlier youth began smartphone ownership. This effect was significant for all groups (8 & under: *b* = −.85, *SE* = .08, *p* < .001; 9 – 10: *b* = -.68, *SE* = .04, *p* < .001; 11-12: *b* = −.44, *SE* = .17, *p* < .001; 13 – 14: *b* = −.27, *SE* = .03, *p* < .001), except for the No Phone group (*b* = −.04, *SE* = .06, *p* = .694). This result suggests that parents reported significantly fewer internalizing problems overall in their children than their children self-reported in all groups, except for in the No Phone group, indicating that reporting discrepancy increased as youth smartphone ownership age decreased. Post-hoc Wald Tests revealed that the informant effect was statistically different for the following comparisons: 9&10 vs. 11&12 (χ2(1) = 26.98, *p* < .001); 11&12 vs. 13&14 (χ2(1) = 5.83, *p* = .015); 13&14 vs. No Phone (χ2(1) = 3.93, *p* = .040).

#### The moderating effect of time on informant discrepancy

Significant interaction (Time × Informant) effects on internalizing problems (see Table 3) were found for all groups except for the No Phone (8 & under: *b* = −.25, *SE* = .05, *p* < .001, 95%CI [−.35, −.15]; 9 – 10: *b* = −.19, *SE* = .02, *p* < .001, 95%CI [−.23, −.14]; 11 – 12: *b* = −.20, *SE* = .01, *p* < .001, 95%CI [−.23, −.16]; 13 – 14: *b* = −.20, *SE* = .04, *p* < .001, 95%CI [−.27, −.14]; No Phone: *b* = −.02, *SE* = .05, *p* = .747, 95%CI [−.12, .09]). These results suggest that parent-child reporting discrepancy increased across time groups where youth owned cell phones. Specifically, parents increasingly reported less youth internalizing problems than their children as participants progressed through the study in these groups. Probing the interaction (see Figure 2 and Table 4) effect in each group revealed there was significant negative informant discrepancy (Youth = 0, Parent = 1) at all timepoints (Y1: *M*_*age*_ = 10.9, Y2: *M*_*age*_ = 10.9, Y3: *M*_*age*_ = 12.9, Y4: *M*_*age*_ = 14.1) for the 8 & under and 9&10 groups. Then, informant discrepancy was significant at Y2-Y4 for the 11&12 group and at Y4 for the 13&14 group. Last, informant discrepancy was not significant at any timepoint for the No Phone group. These results suggest that negative informant discrepancy (i.e., parent-report underestimating youth-report) coincided with age of smartphone use and continued to increase in the years after youth received their first smartphone.

**Table 4.** Probe of the effect of the time × informant product on internalizing problems

		8&under			9&10			11&12			13 & 14			No Phone		
Year	*M*_*age*_	*b*	z	*p*	*b*	z	*p*	*b*	z	*p*	*b*	z	*p*	*b*	z	*p*
1	10.9	−.31	−2.28	.023	−.26	−4.31	< .001	−.01	−.09	.901	.22	.22	.039	.01	.03	.973
2	12.0	−.56	−3.82	< .001	−.45	−6.61	< .001	−.20	−4.14	< .001	.01	.01	.904	−.01	−.06	.952
3	12.9	−.81	−4.67	< .001	−.64	−8.36	< .001	−.39	−6.98	< .001	−.19	−.19	.131	−.03	−.14	.892
4	14.1	−1.05	−5.04	< .001	−.83	−8.98	< .001	−.59	−8.66	< .001	−.40	.40	< .001	−.05	−.19	.848

*Note*. *M*_*age*_ = Mean age at measurement occasion. *b* = the effect of informant on internalizing problems. Significant effects indicate the informant effect is statistically nonzero.

## Discussion

Parents navigate a historically novel and evolving digital context for raising early adolescent youth. A large portion of social interactions transition online as youth progress from childhood through adolescence, with implications for social development. Smartphones provide entertainment and social connectivity that align with adolescents’ changing interests, making ownership highly desirable, especially as peers adopt them. Yet, time tested norms have yet to establish around youth smartphone access and the long-term impacts of early smartphone adoption are not well understood. Evidence is mixed regarding the impact of smartphone use on youth internalizing problems. Some report null effects (Lapierre et al., 2019), while others suggest there are mental health costs associated with smartphone use (Coyne et al., 2019; Wacks & Weinstein, 2021), particularly at higher levels (Liu et al., 2022). Regardless, early detection of internalizing problems is important for preventing further decline, and parents play a central role in this process. The current study provides early evidence that this early detection may be increasingly compromised in early adolescence as youth become smartphone owners at earlier ages, even after controlling for total screen use. First, we showed that informant discrepancy such that parents underestimated youths’ report of their internalizing problems increased across early adolescence across the whole sample. Second, it was more likely that parents underestimated their child’s overall internalizing problems across early adolescence if youth owned smartphones at earlier ages. Then, we also found that this pattern of reporting discrepancy became more prevalent across time for smartphone owners, but not for those who did not own a smartphone. Moreover, parent-child informant discrepancy was statistically significant only in years after youth owned a smartphone.

Consistent with our first hypothesis, our results showed that parents’ underestimation of youth-reported internalizing problems increased across early adolescence in the whole sample. This finding builds on prior cross-sectional empirical evidence demonstrating that parents and adolescent reports often disagree regarding youths’ internalizing problems (De Los Reyes et al., 2015). Compared to other behavior problems, internalizing problems can be more covert making them challenging for parents to observe as they manifest. This is particularly relevant in early adolescence because these types of problems tend to first emerge as a relevant clinical factor at this stage (Blakemore, 2019). Adding to this challenge, early adolescence marks the initiation of substantial changes in the parent-child relationship as youth experience increasing autonomy (Spear & Kulbok, 2004) and reliance on peer support (Brown & Larson, 2009). While these changes are normative and healthy, parents may become less knowledgeable of or slow to recognize changes in their children’s internal state as new relational patterns organize (Granic et al., 2003). Our findings suggest, on average, parents’ underestimation of youths’ internalizing problems often appears at the start of early adolescence and increases in subsequent years. Notably, this pattern for internalizing problems is distinct from findings by Yang et al. (2021) for externalizing problems in which parents reported more symptoms than youth, with informant discrepancy decreasing across early adolescence. To our knowledge, this is among the first longitudinal studies documenting this developmental trend for internalizing problems in this age group.

Results supported our second hypothesis that average parent-child informant discrepancy (i.e., the direct effect of informant on overall internalizing problems) would be more pronounced among youth who acquired smartphones earlier. In all groups except the No Phone group, youth reported significantly higher internalizing problems than their parents. Moreover, informant discrepancy followed a pattern in which the effect was larger in groups characterized by earlier smartphone ownership, respectively. In contrast, no significant informant discrepancy was observed in the No Phone group. This finding suggests that parent-child reporting differences may have a trait-like component that is more pronounced in dyads where youth acquire smartphones earlier.

Smartphone ownership may serve as an indicator of youths’ prior digital media use (i.e., prior to early adolescence), which could contribute to parent-child informant discrepancy. Some supporting evidence suggests digital media use impacts parent-child relationships, which, in turn, influences informant discrepancy (Van Roy et al., 2010). For example, higher youth digital media use has been linked to poorer parent-child relationship quality (Sampasa-Kanyinga et al., 2020), lower attachment quality (Richards et al., 2010), and more reported relationship problems with their parents (Jensen et al., 2021). However, because this pattern persisted after controlling for overall screen time, digital media use alone is unlikely to explain the full effect. Alternatively, early smartphone ownership may reflect broader family technology habits, wherein parents who are heavy digital media users provide phones to their children at younger ages. Indeed, parental smartphone use has been associated with less responsiveness and sensitivity to their children (Abels et al., 2018; Kildare & Middlemiss, 2017). Additionally, family routines may play a role. For example, youth who spend more time away from home may receive smartphones earlier for communication purposes, but reduced parent-child time could limit opportunities for parental monitoring of internalizing problems. In this way, smartphones may be an indicator of broader family dynamics that influence informant discrepancy.

Consistent with our third hypothesis, parent-child informant discrepancy such that parents underestimated youth-reported internalizing problems increased over time in all groups except the No Phone group, even after controlling for total screen time. Also in line with our hypothesis, significant informant discrepancy (i.e., a significant effect of informant on internalizing problems) emerged only after youth acquired a smartphone. Moreover, this discrepancy continued to increase at each subsequent timepoint following smartphone ownership. These findings suggest that group differences in informant discrepancy are linked to smartphone ownership.

Our results indicate that youth smartphone ownership may hinder parents’ ability to detect internalizing problems. Several mechanisms could explain this effect. First, smartphones may facilitate a shift in emotional disclosure from parents to peers. Adolescents primarily use smartphones for social media and texting (Alexander et al., 2024), granting them continuous access to an online social network that other media do not. As youth increasingly seek peer support during adolescence (Brown & Larson, 2009), smartphones enable youth to confide in peers at any time and place, possible reducing their frequency of disclosure to parents. In this way, smartphones may act as a barrier to parental awareness of youth internalizing concerns.

Youth smartphone use may not only shift disclosure patterns but also influence the parent-child relationship more broadly, with implications for closeness and communication. At proximal time scales, smartphone use can disrupt interactions and shared activities through notifications and frequent checking (McDaniel, 2020). Additionally, parents often cite smartphones as a source of conflict when enforcing technology boundaries (Hattersley et al., 2009). Over time, these events may accumulate, altering the dynamics of the parent-child dyad. Furthermore, earlier smartphone ownership has been linked to greater aggressiveness and irritability (Thiagarajan & Newson, 2025), potentially making interactions more challenging and increasing friction in the relationship. As a result, reduced closeness may mediate the relation between early smartphone ownership and informant discrepancy. If so, the effects of early smartphone adoption may extend beyond disclosure patterns to the broader parent-child relationship itself. Future research should examine whether smartphone ownership age contributes to long-term changes in parent-child dynamics.

Consistent with prior research, we found that most youth acquired their first smartphone by age 12, with few remaining without one by age 14. This stresses the normative role of smartphones in adolescent development and the importance of considering their impact during this stage. Additionally, we identified demographic differences in smartphone ownership trends. Girls and youth with less-educated parents were more likely to acquire smartphones earlier. These findings align with prior research showing that girls use smartphones more frequently than boys (Twenge & Martin, 2020) and that parents with higher education levels tend to impose more digital media restrictions (Livingstone et al., 2015).

Although this study does not establish causality between smartphone ownership and internalizing problems, one notable trend warrants discussion. Across all smartphone-owning groups, youth who acquired smartphones earlier consistently reported higher levels of internalizing problems at each timepoint. This pattern suggests an underlying process linking smartphones and internalizing problems in early adolescence. Several explanations are possible. Smartphone ownership may serve as a marker of an unmeasured factor, such as family or peer influences, that simultaneously promotes early smartphone adoption and increases internalizing problems risk. Alternatively, youth experiencing psychological distress may advocate for smartphone ownership earlier. In support, prior research shows increased screen use among individuals with depression, potentially using screens as a coping mechanism (Elmquist & McLaughlin, 2018; Wolfers & Utz, 2022). Conversely, smartphone use itself may contribute to internalizing problems (Twenge, 2020). Overall, empirical studies in middle adolescence suggest that problematic (e.g., smartphone dependency) and high levels of smartphone use tends to be associated cross-sectionally and longitudinally with increased internalizing problems (Augner et al., 2021; Lapierre et al., 2019; Ng et al., 2020; Wacks & Weinstein, 2021). Yet, null results (Poulain et al., 2023) and even contrary findings showing mental health benefits from using smartphones (Marciano et al., 2022; Minich & Moreno, 2024) are also present. Our results highlight the need for more longitudinal studies on this link, particularly in early adolescence, and provide a more nuanced reason for negative associations in that they may create barriers to identifying symptoms.

Our findings indicate that the No Phone group differed from smartphone users in several key ways. First, prior research consistently shows that parents report lower levels of internalizing problems than youth self-report (De Los Reyes et al., 2015). This pattern held across all groups except the No Phone group, where parent and child reports were highly aligned. Second, latent growth analyses revealed that while internalizing problems significantly increased in all smartphone groups, they decreased over time in the No Phone group. Third, parent-reported internalizing problems were consistently higher in the No Phone group than in smartphone groups. However, because parents of smartphone users tended to underestimate youth-reported internalizing problems, parent reports in the No Phone group were the most similar to youth self-reports. These findings suggest that the No Phone group may represent a distinct population. Research on this form of smartphone abstention is scarce and given that this group was a small minority in our sample, their divergence from the normative trend of early smartphone adoption warrants further investigation. Future studies should explore the family, peer, and individual factors associated with delaying smartphone ownership.

### Limitations

These findings should be considered in light of several limitations. First, while we controlled for overall screen use at each timepoint, we could not account for the frequency of smartphone use throughout the study. Future research should examine how smartphone use, beyond ownership, influences parent-child informant discrepancy. Second, smartphone behaviors vary widely among youth. If primarily used for parental communication, smartphones might enhance rather than hinder parent-child agreement. Future studies should distinguish between different usage patterns to identify risks and benefits. Third, our focus was on informant discrepancy in internalizing problems. It remains unclear whether similar patterns extend to other constructs, such as externalizing behaviors or family dynamics. Fourth, parents retrospectively reported the age of smartphone acquisition, and internalizing problems were assessed at one-year intervals. Future research should measure smartphone adoption more precisely and examine informant discrepancy at shorter timescales before and after acquisition to capture more proximal effects. Moreover, most parents in the current study were mothers. Although mother-father agreement regarding youth internalizing problems tends to be high (Schroeder et al., 2010), prior study results suggest there are differences between mother- and father-child reporting discrepancy. However, consistent patterns have yet to emerge as some studies have shown better child agreement with fathers (Hughes & Gullone, 2010; Treutler & Epkins, 2003), and others with mothers (Seiffge-Krenke & Kollmar, 1998). Future studies should test whether findings from the current study differ between father- and mother-reports. Further, although we demonstrated that smartphone ownership is associated with reporting discrepancy, other sources of reporting discrepancy should be explored in future studies (e.g., autonomy development and parental monitoring). Finally, youth-reported internalizing problems were successively higher in groups with earlier smartphone ownership, making it difficult to disentangle informant discrepancy from overall group differences in internalizing problems. However, our multi-level modeling approach accounts for between- and within-person variance and mitigates such confounding issues inherent to difference scores (De Los Reyes et al., 2016; Laird & De Los Reyes, 2013). Nonetheless, future research should replicate these findings in clinical samples to further clarify these relations.

### Conclusion

Despite these limitations, our study leverages advanced statistical methods and a large, longitudinal, and generalizable dataset to examine parent-child informant discrepancy in youth internalizing problems. First, we found that overall discrepancy was more pronounced among youth who received smartphones earlier, suggesting that studies relying on parent-reported internalizing problems should account for youth smartphone ownership. Second, informant discrepancy increased over time among smartphone owners, highlighting a potential long-term effect of smartphone use on parent-child agreement regarding internalizing problems. Finally, youth without smartphones exhibited distinct developmental patterns in internalizing problems based on both parent- and self-reports, underscoring the need for further research on this subgroup. Our findings provide important insights into the role of smartphone ownership in adolescent psychosocial development and suggest early smartphone ownership may lead to parents’ underestimation of their children’s internalizing problems in early adolescence.

## Supporting information

10.1017/S0954579425100618.sm001 Carvalho et al. supplementary material Carvalho et al. supplementary material

## Data Availability

The data that support the findings of this study are available to qualified researchers with a valid data use contract in the NIMH Data Archive (NDA) at https://nda.nih.gov, NDA Collection #2573, DOI: 10.15154/z563-zd24.

## Supplementary material

The supplementary material for this article can be found at https://doi.org/10.1017/S0954579425100618.
