# Supplementary material for: The role of smartphones in adolescent-parent discrepancy in reporting adolescents’ internalizing problems
Source: Dev Psychopathol. 2025 Sep 8:1–13. Online ahead of print. doi: 10.1017/S0954579425100618 (PMC12752471; doi:10.1017/S0954579425100618)
Supplement: Carvalho et al. supplementary material [file S0954579425100618sup001.docx]

**Supplemental Figure 1**

*Histograms showing the distributions of parent and child reported internalizing problems at each timepoint.*


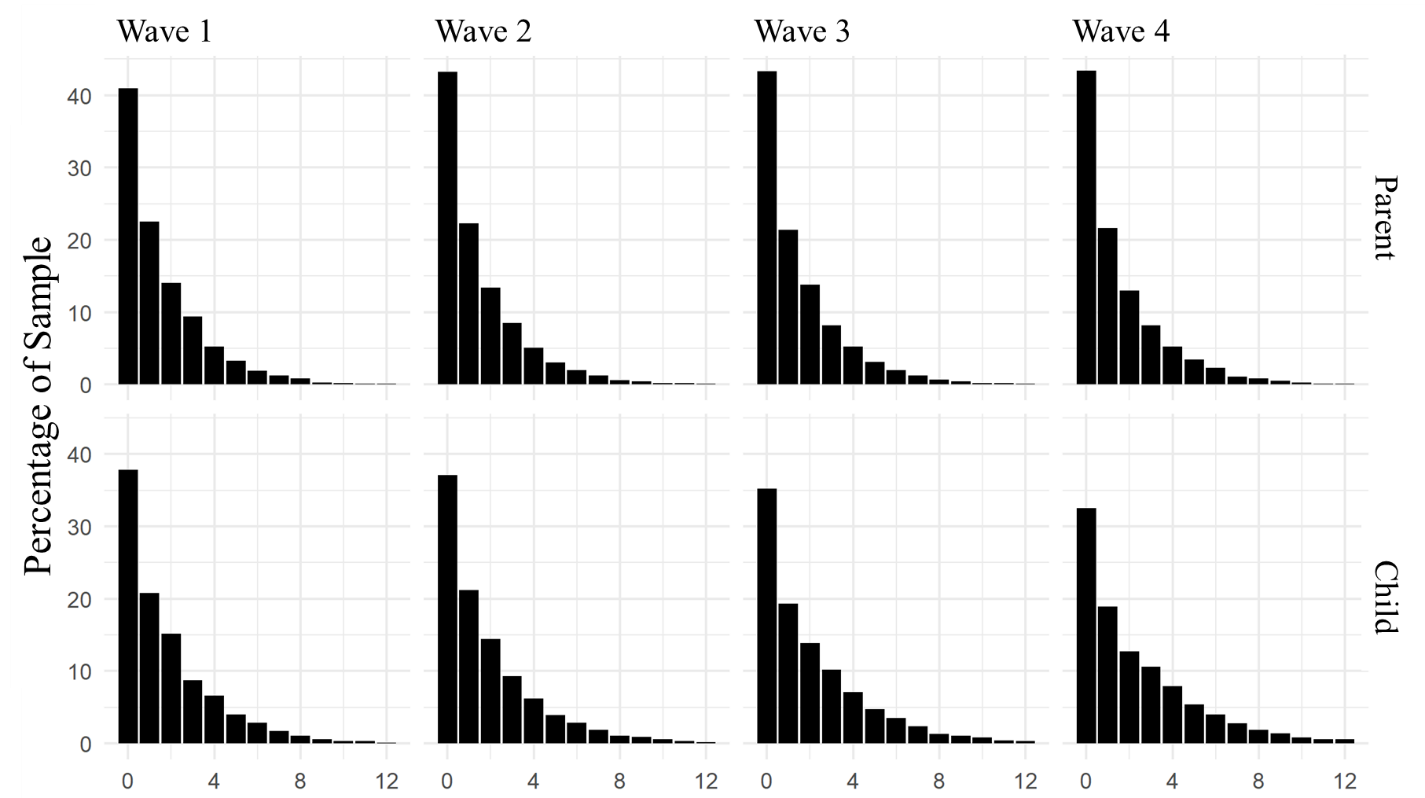


**Supplemental Table 1**

*Table showing the 6 items shared between the Brief Problem Monitor (youth-report) and Child Behavior Checklist (parent-report).*

| Item text |
| --- |
| Feels worthless or inferior |
| Too fearful or anxious |
| Feels too guilty |
| Self-conscious or easily embarrassed |
| Unhappy, sad, or depressed |
| Worries |
